# Development of an LC–TOF/MS Method to Quantify Camrelizumab in Human Serum

**DOI:** 10.3390/molecules29204862

**Published:** 2024-10-14

**Authors:** Li Song, Yan Liang, Yilin Li, Tingting Guo, Hui Li, Shuxuan Liang

**Affiliations:** 1 College of Chemistry and Materials Science, Hebei University, Baoding 071000, China; 2 College of Pharmacy, Hebei Medical University, Shijiazhuang 050000, China; 3 College of Chemistry and Pharmaceutical Engineering, Hebei University of Science and Technology, Shijiazhuang 050000, China; 4 Hebei Institute of Drug and Medical Device Inspection, Shijiazhuang 050000, China

**Keywords:** camrelizumab, LC-TOF/MS, serum, PD-1 inhibitor, monoclonal antibody

## Abstract

With the advantages of a high specificity, a long half-life, and a high safety, the use of antibody biologic drugs, including camrelizumab, has been rapidly increasing in clinical practice. Camrelizumab, an immune checkpoint inhibitor and humanized monoclonal antibody, is used to treat several advanced solid cancers. Measuring its concentration supports personalized dosage adjustments, influences treatment decisions for patients, strengthens the control of disease activity through therapeutic drug monitoring, and helps evaluate and prevent drug interactions in combination therapy. Because antibodies are present in complex biological matrices, quantifying monoclonal antibody drugs is challenging, and must rely on precise, selective, and reliable analytical methods. In this study, a quadrupole time-of-flight mass spectrometry TripleTOF 6600+ (AB SCIEX, Framingham, MA, USA) system equipped with a Turbo V ion source was used for the qualitative analysis of monoclonal antibodies using the data-dependent acquisition (IDA) MS/MS mode, followed by quantitative analysis using a targeted MRMHR workflow. This method showed a good linear relationship within the range of 4–160 μg/mL, with a correlation coefficient of R^2^ ≥ 0.996. It demonstrated an acceptable accuracy (88.95–101.18%) and precision (≤15%). Furthermore, the lower limit of quantification was found to be 4 μg/mL, with the lowest detection limit of 0.3217 μg/mL, indicating that this method is rapid, accurate, and reliable for the quantitative analysis of camrelizumab in human serum.

## 1. Introduction

Hepatocellular carcinoma (HCC) is a malignant tumor with a high incidence observed in China, which has one of the highest rates of HCC worldwide. HCC often has an insidious onset and a lack of specific clinical manifestations, resulting in a low rate of early diagnosis. This phenomenon frequently leads to progression to advanced stages, and is associated with a poor prognosis and high mortality [1,2]. Currently, liver cancer is primarily treated with radiation therapy, liver transplantation, targeted therapy, and immunotherapy. Tumor immunotherapy primarily depends on activated T cells to target and kill tumor cells. However, programmed cell death protein 1 (PD-1), a receptor on T cells, serves as an immune checkpoint by binding with programmed death ligand 1 (PD-L1) on cancer cells, leading to an immunosuppressive effect. Therefore, immunotherapy with checkpoint inhibitors is commonly used in clinical practice for tumor treatment, with PD-1 antibody being the most successful type [3,4,5]. These antibodies activate inactive T cells, enabling them to effectively target and eliminate tumor cells.

Camrelizumab (SHR-1210) is an immune checkpoint inhibitor, a humanized IgG4-κ anti–PD-1 monoclonal antibody (mAb), that binds to PD-1 with a high affinity. This binding retards the interaction between PD-1 and PD-L1, thereby exerting its immunotherapeutic effects. Camrelizumab has been reported to be effective in the treatment of various cancers, including nonsmall cell lung cancer [6,7,8], esophageal squamous cell carcinoma [9,10,11], Hodgkin’s lymphoma [12,13,14], and advanced HCC [15]. Furthermore, it is anticipated to be an effective treatment for various malignancies. Several studies have reported the effectiveness of camrelizumab in patients with advanced HCC [16,17,18,19]. For example, one study reported that combining camrelizumab with sorafenib, transarterial chemoembolization (TACE), and radiotherapy to treat portal vein tumor thrombosis in patients with advanced HCC resulted in a median progression-free survival (PFS) of 15.7 months and a 1-year overall survival (OS) of 83.3% [19]. Another study observed that the combination of camrelizumab and lenvatinib achieved a median PFS of 8.0 months in patients with advanced HCC, which was higher than that of patients treated with lenvatinib alone [16]. Ran You et al. found that camrelizumab, when combined with a TACE regimen, was both effective and safe, suggesting its potential as a therapeutic option for the treatment of patients with intermediate-to-advanced HCC [18]. However, camrelizumab can induce adverse reactions such as hepatic cavernous hemangioma and anaphylactic shock, necessitating the rapid and accurate monitoring of the drug in the blood [20,21].

Currently, detection methods for serum camrelizumab levels are limited and largely depend on immunoassay techniques. However, these methods have certain limitations, including a narrow measurement range, poor precision, lengthy processes for obtaining specific antigens or antibodies, and susceptibility to matrix interference [22,23]. As an emerging analytical method, mass spectrometry (MS) offers a wide dynamic range and can rapidly and accurately detect analyte signals in complex biological matrices, providing a high sensitivity, excellent accuracy, and strong selectivity. Aurélien Millet found no significant difference in the quantification of nivolumab in human serum using liquid chromatography (LC)-MS and enzyme-linked immunosorbent assays; however, MS was found to be a more facile and sensitive technique [24]. Researchers have developed and validated LC-MS/MS methods for the simultaneous quantification of trastuzumab and pertuzumab, establishing a foundation for quantifying mAb drugs [25]. Furthermore, adalimumab, tocilizumab, and infliximab have been successfully quantified in serum using LC-MS/MS [26,27,28].

To the best of our knowledge, there are no existing reports on the quantification of camrelizumab using LC-MS. This study introduces a method for the quantitative analysis of camrelizumab in serum by leveraging advanced mass spectrometry techniques.

## 2. Results

### 2.1. Calibration Curve

A linear regression analysis of the peak area of camrelizumab was performed using eight serum samples with varying concentrations (4, 10, 15, 20, 40, 80, 100, and 160 μg/mL). The linear equation showed a linear correlation coefficient R^2^ of 0.9967 and a weight of 1/*X*. The lowest concentration was 4 μg/mL and the lowest limit of detection was 0.3217 μg/mL, demonstrating that the developed method is both sensitive and suitable for quantitative analysis.

### 2.2. Selectivity and Carryover

**Figure 1:**
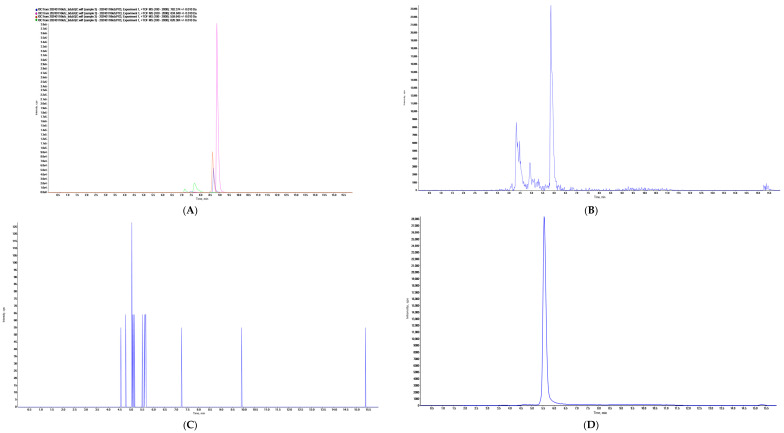
(**A**): XIC map of the 4 candidate peptides, (**B**): XIC map of TTPPVLDSDGSFFLYSR in blank serum, (**C**): XIC map of LLIYTATSLADGVPSR in blank serum, (**D**): XIC map of LLIYTATSLADGVPSR in serum sample.

The specificity of target peptides in six blank serum samples was investigated. As shown in Figure 1, the characteristic peptides demonstrated specific signals without interference. After three independent injections of the upper limit of quantitation samples, the signal of the blank serum sample was determined to be <20% of the lower limit of quantification (LLOQ) signal.

### 2.3. Precision and Accuracy

Intraday and interday precision and accuracy of quality control (QC) samples (LLOQ: Lower Limit of Quantification, LOQ: Low Quality Control, MOQ: Middle Quality Control, HOQ: High Quality Control) (*n* = 6) are shown in Table 1. Intraday and interday precision, assessed over three consecutive days, were both observed to be below 9%, with the precision for the daytime LLOQ group being under 15%. Accuracy ranged from 88.95% to 101.18%. These results confirm that the developed method meets the validation criteria for precision and accuracy, demonstrating its reliability and reproducibility.

### 2.4. Stability

The stability test results showed that the camrelizumab serum samples remained stable under various conditions: at 4 °C for 24 h, at 25 °C for 24 h, after being repeatedly frozen and thawed three times, and at −80 °C for 3 months. The results are shown in Table 2.

### 2.5. Matrix Effects and Recovery Rates

As shown in Table 3, the recovery rate of camrelizumab serum samples at low, medium, and high concentrations was determined in the range of 82.07–93.23% with an RSD ≤ 7.68%. The matrix effect was 75.83% at low concentration and 77.17% at high concentration. Although the method could not eliminate the matrix effect due to the complexity of the matrix, it did not influence the accuracy or precision of the results, and the recovery rates were found to be satisfactory.

### 2.6. Dilution Reliability

A 5-fold dilution verification test was conducted by diluting a high-concentration serum sample of 650 μg/mL to 130 μg/mL (HOQ), *n* = 4. The 5-fold diluted sample showed an accuracy of 89.29% and an RSD of 3.66%. These results suggest good precision and accuracy, demonstrating the reliability of the method even after a 5-fold dilution of the sample.

### 2.7. Robustness

Robustness is the ability to measure the ability to be unaffected by small intentional changes in method parameters. The durability of the method was analyzed by changing the flow rate of the mobile phase and the column temperature. A standard solution of 40 μg/mL was injected into the liquid chromatography–mass spectrometer, and the flow rate was adjusted from 0.3 mL/min to 0.29 mL/min and 0.31 mL/min, respectively; the column temperature was changed from 40 °C to 39 °C and 41 °C, and the content was measured at each setting. The results showed that the values measured before and after changing the flow rate and initial column temperature were essentially consistent, proving that the method has a good robustness.

## 3. Discussion

This study developed an LC-TOF/MS method for quantifying camrelizumab concentrations in human serum. Serum samples were initially precipitated with methanol and then resuspended in ammonium bicarbonate to reduce sample complexity. Reductive and alkylating agents were then used to denature the protein, followed by trypsin digestion to obtain characteristic peptide segments of camrelizumab. A phase I dose escalation study on camrelizumab in advanced solid tumors (NCT02742935) showed that the peak concentrations (C_max_) of the 60, 200, and 400 mg camrelizumab groups were observed at 20.0, 70.4, and 127 μg/mL, respectively [29]. The results indicated that the quantitative range of this experiment covers the clinical concentration of camrelizumab. Monitoring these concentrations is essential for assessing and preventing drug interactions in combination therapies, and adjusting doses for individual patients, and aligns with the purpose of therapeutic drug monitoring (TDM).

However, this study still possesses certain limitations. Although the quantitative range of this method covers the clinical concentrations reported for camrelizumab and the validation results meet FDA regulatory guidelines [30], its application in clinical samples has not yet been elucidated. Kei Irie et al. successfully developed and validated an LC-MS/MS absolute quantification method for the determination of nivolumab. Combined with PPK analysis, this method can be used for clinical TDM analysis of nivolumab [31]. Currently, there are no existing reports on the use of LC-MS for the absolute quantification of camrelizumab in human serum.

In summary, monitoring camrelizumab levels in human serum is of great clinical significance.

## 4. Materials and Methods

### 4.1. Reagents and Instruments

Camrelizumab (146.3 KD) was purchased from MCE. Formic acid (LC-MS, 99% purity) was purchased from Shanghai Aladdin Biochemical Technology Co., Ltd. (Shanghai, China). Methanol and acetonitrile (HPLC grade, ≤100% purity) were obtained from Thermo Fisher Technology Co., Ltd. (Shanghai, China). Dithiothreitol (DTT, 99.5% purity, molecular weight [MW] 154.25) and iodoacetamide (IAM, ≥99% purity, MW 184.96) were purchased from Shanghai Solarbio Bioscience & Technology Co., LTD. (Shanghai, China). and Shanghai Macklin Biochemical Technology Co., Ltd. (Shanghai, China)., respectively. Trypsin was purchased from Sigma Corporation, Germany (Osterode, Germany). Ultrapure water (resistivity 18.2 mΩ cm) was obtained using a MilliQ Plus^®^ system (Millipore, Molsheim, France). High-purity nitrogen (>99.9% purity) was obtained from Shanghai, while other reagents were prepared in our laboratory. Low-adsorption polypropylene microtubules used in this study were purchased from Dutsher (Brumas, France).

Triple TOF 6600+ (AB SCIEX, Framingham, MA, USA) and the high-speed refrigerated centrifuge were purchased from Sigma (Osterode am Harz, Germany), the vortex oscillator was purchased from IKA (Staufen, Germany), the electronic scale was procured from Huazhi Electronic Science and Technology Co., Ltd. (Fujian, China), the pH acidometer was purchased from INESA Scientific Instrument Co., Ltd. (Shanghai, China), and a thermostatic water bath was obtained from Memmert (Schwabach, Germany).

### 4.2. Chromatographic and MS Conditions

#### 4.2.1. Chromatographic Conditions

Chromatography was performed using Agilent Poroshell 120 EC-C18 columns (2.1 × 100 mm, 1.9 µm). The column temperature was maintained at 40 °C while the temperature of the sample tray was set at 4 °C. Mobile phase (A) comprised 0.1% (*v*/*v*) formic acid in water, and mobile phase (B) was based on acetonitrile.

The gradient elution procedure was as follows: 0–1 min at 95% A, 1–7 min from 95 to 55% A, 7–9 min from 55 to 25% A, 9–11 min from 25 to 10% A, 11–14 min at 10% A, 14–14.1 min from 10 to 95% A, 14.1–16 min from 95% A. The total analysis time was 16 min, with an injection volume of 5 µL, and a flow rate of 0.3 mL/min.

#### 4.2.2. MS Conditions

All analytes were analyzed by mass spectrometry using electrospray ionization (ESI) in positive ion mode and multiple reaction monitoring (MRM) with Analyst^®^ TF 1.8.1 and SCIEX OS 1.5.0.23389 software for data acquisition and processing. The ion source parameters were adjusted as follows: ion source temperature at 500 °C, ion spray voltage at 5500 V, ion source gas 1 and gas 2 at 60 psi each, declustering potential at 80 V, collision energy at 25 V, and curtain gas at 30 psi. Moreover, the analysis was performed in peptide mode.

### 4.3. Solution Preparation

#### 4.3.1. Preparation of Experimental Reagents

We dissolved 100 mM NH_4_HCO_3_: 1.581 g NH_4_HCO_3_ (MW 79.06) in 200 mL MilliQ water, pH = 8.

We dissolved 100 mM DTT: 0.0154 g DTT (MW 154.3) in 1 mL of 100 mM NH_4_HCO_3_ solution.

We dissolved 200 mM IAM: 0.0370 g IAM (MW 185.0) in 1 mL of 100 mM NH_4_HCO_3_ solution.

Trypsin solution: The solution was digested with trypsin in 1 mM hydrochloric acid at a concentration of 1 mg/mL (20 μg trypsin and 20 μL 1 mM hydrochloric acid), pH = 3.

#### 4.3.2. Preparation of Sample and Standard Solutions

Blood samples from healthy volunteers were collected in blood collection tubes. The blood samples were centrifuged at 4000 r/min for 15 min at 4 °C to obtain the supernatant, which was then stored at −80 °C until use. The study was approved by the Ethics Committee of Hebei Medical University.

A volume of 500 μL of normal saline was added to camrelizumab powder to obtain a stock solution of 2 mg/mL. The stock solution (5 μL) was transferred into a 1.5 mL centrifuge tube and serially diluted with 2495, 995, 660, 495, 245, 120, 95, and 57.5 μL serum, respectively, to prepare standard samples with concentrations of 4, 10, 15, 20, 40, 80, 100, and 160 μg/mL. A total of 25 µL of serum sample was precisely aspirated, and proteins were precipitated with 100 µL of methanol before being stirred for 2 min and centrifuged at 17,000× *g* for 20 min. The resulting precipitate was transferred to an Eppendorf (EP) tube. Next, the protein precipitate was resuspended with 200 μL NH_4_HCO_3_ (pH = 8, 100 mM) by vigorously vortexing for 2 min until a uniform white protein suspension was obtained. An appropriate amount of the reducing agent dithiothreitol (DTT) was added to achieve a final concentration of 5 mM. The disulfide bonds were cleaved by incubating the mixture for 30 min at 56 °C. After cooling the samples at 25 °C, an appropriate amount of the alkylating agent iodoacetamide (IAM) was added to yield a final concentration of 10 mM and the mixture was incubated for 15 min in the dark to denature the sulfhydryl groups. Lastly, 100 μL of pancreatic enzyme solution was added and incubated overnight for 12 h at 37 °C. The enzymatic reaction was then terminated by adding 2 μL of formic acid. The mixture was then centrifuged at 17,000× *g* for 20 min, and 100 μL of the supernatant was collected in a clean EP tube for analysis.

### 4.4. Optimization of the Experimental Process

#### 4.4.1. Optimization of DTT and IAM Concentrations

**Figure 2:**
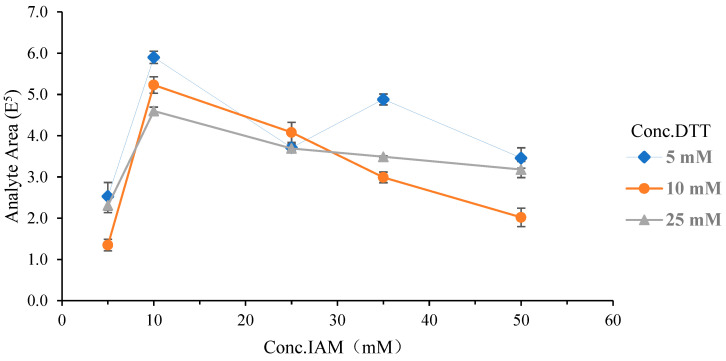
Influence of different concentrations of DTT/IAM on the signal response of the target peptide.

MAbs are complex macromolecular glycoproteins with intricate folded structures. To ensure effective enzymolysis, the proteins must be fully denatured before incubation. DTT was used to reduce the encapsulated disulfide bonds, generating free sulfhydryl groups. Subsequently, IAM was added to alkylate these sulfhydryl groups, preventing the reformation of stable disulfide bonds. The concentrations of DTT and IAM significantly influence the efficiency of enzymatic hydrolysis. In this research, a two-factor cross-test was conducted to determine the optimal experimental concentrations of both DTT and IAM. The optimal concentrations of DTT were identified as 5, 10, and 25 mM, while the optimal concentrations of IAM were determined to be 5, 10, 25, 35, and 50 mM (Figure 2). At 5–10 mM concentrations of IAM, the peptide response values increased with rising IAM concentration across different DTT levels. When the IAM concentration reached 10 mM, the value of signal response peaked under various DTT concentrations. Beyond this point, increasing the IAM concentration led to a gradual decrease in signal response at DTT concentrations of 10 and 25 mM. The signal response increased as the IAM concentration increased from 25 to 35 mM at a DTT concentration of 5 mM, but it did not reach the maximum value. After that, the response value of the peptide decreased with an increase in IAM concentration. Therefore, the optimal concentration of DTT/IAM was determined to be 5 mM/10 mM.

#### 4.4.2. Optimization of Enzymatic Hydrolysis Time

**Figure 3:**
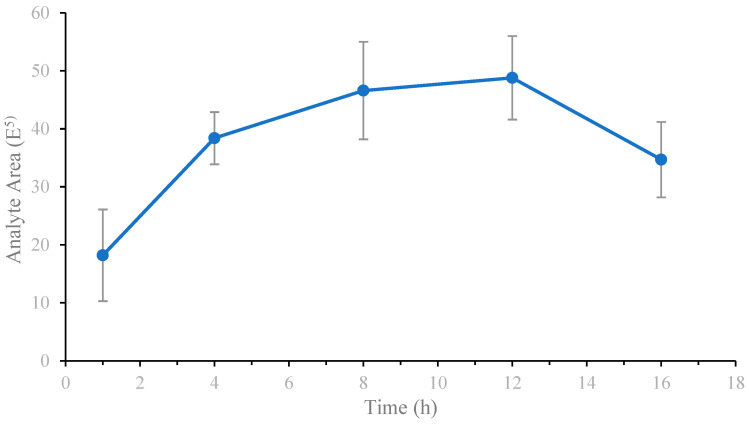
Signal response of the target peptide segment at different enzymolysis times (*n* = 3).

Enzymolysis efficiency was determined using peak area measurements. Figure 3 shows that the enzymolysis efficiency of the pancreatic enzyme gradually increased during the first 1–8 h. The increasing trend slowed down during 8–12 h and the enzymolysis efficiency decreased after 12 h. Considering the experimental data and time constraints, the enzymatic hydrolysis method with 12 h of overnight incubation was selected.

#### 4.4.3. Selection of Quantitative Ion Pairs and Optimization of MRM Parameters

**Figure 4:**
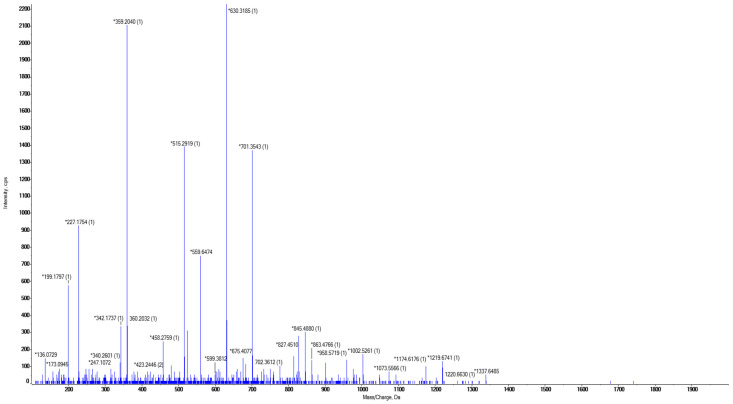
Secondary mass spectra of the peptide LLIYTATSLADGVPSR. *: The *m*/*z* of daughter ion.

First, the amino acid sequence of camrelizumab was obtained from the IMGT website. Enzyme digestion prediction was performed using PeptideMass and Skyline. Based on the predicted results, the peptides GLEWVATISGGGANTYYPDSVK, TTPPVLDSDGSFFLYSR, LLIYTATSLADGVPSR, and EPQVYTLPPSQEEMTK were selected as candidate peptides. The mass-to-charge (*m/z*) ratio and the charge number of the candidate peptides are shown in Table 4. Their specificity and response strength were determined to evaluate their suitability for quantification. Figure 1A–D illustrates that the TTPPVLDSDGSFFLYSR showed a high response; however, its applicability is restricted by the substantial interference from blank serum samples. The peptide LLIYTATSLADGVPSR demonstrated a distinct peak with high response and no interference in blood samples, making it suitable as a characteristic peptide. The daughter ion fragments of this peptide are illustrated in Figure 4. The MS MRM^HR^ method was used to screen 5 of these ion pairs and select the best quantitative ion pairs. During this process, parameters such as declustering potential (DP) and collision energy (CE) were continuously optimized to increase the signal response. Finally, the ion pair *m/z* 559.64544 [M + 3H]^3+^/359.20370 [M + 1H]^+^ with a high signal response and stable signal was selected as the quantitative ion pair with DP value of 80 V and CE value of 25 V (Table 5, Table 6 and Table 7).

Furthermore, the mobile phase and columns were optimized.

### 4.5. Method Validation

The assay was thoroughly validated following EMA and FDA guidelines for bioanalytical method validation. The evaluation process included the following parameters: linearity, accuracy and precision, specificity, matrix effect, recovery, dilution reliability, robustness and stability. The calibration curves of the eight serum standards (4, 10, 15, 20, 40, 80, 100, and 160 μg/mL) were established using an LC-TOF/MS method. A weighted (1/*X*) linear least-squares regression method was used to construct a standard curve, plotting sample concentration on the *x*-axis and camrelizumab peak area on the *y*-axis. Six blank serum samples from various sources were processed according to the standard protocol of blood serum sample preparation and analyzed to identify any specific disturbances or interferences. Quality control (QC) samples, labeled as low QC (LQC), medium QC (MQC), and high QC (HQC) were prepared in blank human serum at concentrations of 10, 80, and 130 μg/mL, respectively. The intraday and interday accuracy and precision of the method were evaluated by analyzing six QC samples at the lowest concentration (LLOQ), as well as at low, medium, and high concentrations, across three consecutive analytical batches. The interday and intraday precision of the low, medium, and high concentrations should be ≤15%, while the LLOQ concentration should be ≤ 20%. The accuracy should be within ±15% of the labeled value of the QC samples, and the LLOQ should be within ±20% of the labeled value.

The stability of the method was assessed by distributing the configured samples into five groups: immediate injection analysis; storage at 4 °C for 24 h before analysis; storage at room temperature for 24 h before analysis; preserve at −80 °C for three months; and three freeze–thaw cycles at −80 °C, with each freeze lasting over 10 h. To determine the matrix effect, new QC samples at low and high concentrations were prepared and analyzed for precision and accuracy in both serum and NH_4_HCO_3_ diluents. Furthermore, the recovery rate was assessed at low, medium, and high concentrations. Since the mAb drug concentrations in patient serum are typically high, the method was also tested with 5-fold diluted samples (*n* = 4) to ensure that dilution did not compromise precision or accuracy.

## 5. Conclusions

A new LC-TOF/MS bioanalytical method with high-resolution tandem MS has been developed and validated. This method effectively identified and quantified samples with a wide and dynamic concentration range, allowing a rapid and comprehensive analysis. The developed method showed a good stability under various conditions. Despite certain matrix effects being observed, it achieved good recovery rates, high precision, and accuracy. Therefore, this method can be effectively used to quantify camrelizumab in human serum and may also serve as a novel approach to determine the concentrations of other monoclonal antibodies in blood samples.

## Figures and Tables

**Table 1:** Results of QC samples intraday and interday precision and accuracy (*n* = 6).

Concentration (μg/mL)		Precision (%)		Accuracy (%)	
Spiked (μg/mL)	Found (μg/mL) (Mean ± SD)	Intraday	Interday	Intraday	Interday
4 (LLOQ)	3.68 ± 0.33	8.93	14.56	91.95	95.34
10	10.19 ± 0.81	8.03	8.16	101.18	93.66
80	75.62 ± 5.82	7.69	5.89	94.52	92.32
130	115.64 ± 3.13	2.87	2.90	88.95	89.59

**Table 2:** Stability statistics of analytes in different conditions (*n* = 6).

QC Levels	LQC (10 μg/mL)		MQC (80 μg/mL)		HQC (130 μg/mL)	
Stability Tests	RSD (%)	RE (%)	RSD (%)	RE (%)	RSD (%)	RE (%)
Condition	*n* = 6					
at once	6.70	2.48	2.49	9.80	7.20	−6.20
4 °C/24 h	8.65	1.81	4.73	8.36	3.01	−0.58
25 °C/24 h	7.78	5.33	7.40	7.09	8.87	−6.76
3 Freeze–thaw cycles	7.40	0.70	3.24	8.54	8.09	−4.36
−80 °C/3 months	8.58	8.50	1.64	5.64	5.41	−5.97

RE: relative error. RSD: relative standard deviation.

**Table 3:** Recovery and matrix effect of camrelizumab in human serum (*n* = 6).

Spiked (μg/mL)	Recovery (%)		Matrix Effect (%)
	Mean	RSD	Mean
10	92.47	4.00	75.83
80	82.07	7.68	
130	93.23	4.44	77.17

**Table 4:** *m*/*z* and charges of the candidate peptides.

Compound	Selected Peptide	Precursor Ion	
		(*m/z*)	Charge
Camrelizumab	GLEWVATISGGGANTYYPDSVK	762.37434	+3
	TTPPVLDSDGSFFLYSR	634.64751	+3
	LLIYTATSLADGVPSR	559.64544	+3
	EPQVYTLPPSQEEMTK	626.30500	+3

**Table 5:** The area under the peak of the different daughter ions.

Compound	Precursor Ion (*m/z)*	Daughter Ion (*m/z*)	Ion	Peak Area
		701.3577	y7	1.13 × 10^5^
		630.3206	y6	1.61 × 10^5^
Camrelizumab	559.64544	515.2936	y5	1.14 × 10^5^
		359.2037	y3	1.89 × 10^5^
		340.2595	b3	1.90 × 10^4^

**Table 6:** DP value and peak area of the target peptide in the MRM mode.

Analyte	Amino Acid Sequence	MRM Ion Transition, *m/z*	Declustering Potential (V)	Peak Area
			45	2.76 × 10^5^
Signature peptide	LLIYTATSLADGVPSR	559.64544 (3+) >359.2037 (1+)	80	5.79 × 10^5^
			90	2.59 × 10^5^

**Table 7:** CE value and peak area of the target peptide in the MRM mode.

Analyte	Amino Acid Sequence	MRM Ion Transition, *m/z*	Declustering Potential (V)	Collision Energy (V)	Peak Area
				15	2.56 × 10^4^
				20	2.11 × 10^4^
Signature peptide	LLIYTATSLADGVPSR	559.64544 (3+) >359.2037 (1+)	80	25	2.77 × 10^4^
				30	2.65 × 10^4^
				35	1.24 × 10^4^
				40	1.66 × 10^4^

## Data Availability

Data are contained within the article. The original contributions presented in the study are included in the article, further inquiries can be directed to the corresponding authors.
